# Neuromorphic-inspired multi-view global-local fusion for IR-UWB radar dynamic gesture recognition

**DOI:** 10.3389/fnins.2026.1864448

**Published:** 2026-06-19

**Authors:** Guoyi Xue, Junhong Yang, Hui Zhu

**Affiliations:** School of Engineering Science, Shandong Xiehe University, Jinan, China

**Keywords:** dynamic gesture recognition, feature fusion, IR-UWB radar, MULTI-view sensing, time-range maps

## Abstract

**Introduction:**

Dynamic gesture recognition using impulse radio ultra-wideband (IR-UWB) radar has attracted increasing interest for privacy-preserving and illumination-robust human-computer interaction. However, single-view radar perception is susceptible to occlusion and viewpoint-dependent information loss, while existing methods often struggle to jointly model fine-grained local motion patterns and long-range temporal dependencies in time-range (TR) representations.

**Methods:**

To address these issues, this paper proposes a neuromorphic-inspired multi-view global-local fusion network for IR-UWB radar dynamic gesture recognition. Specifically, motion-enhanced TR maps from three complementary viewpoints are first integrated via early fusion to improve the spatial completeness of radar observations. A dual-branch architecture is then employed to capture local dynamic textures and global temporal structures in parallel. In addition, an adaptive fusion module combining gated first-order fusion and bilinear second-order interaction is introduced to enhance feature complementarity and representation discriminability.

**Results:**

Experiments on a public 12-class UWB gesture dataset under a subject-independent protocol show that the proposed method achieves an average accuracy of 98.29%, outperforming several representative baselines.

**Discussion:**

These results demonstrate the effectiveness of the proposed framework for robust multi-view radar-based dynamic gesture recognition.

## 1 Introduction

Human–computer interaction (HCI) is increasingly evolving from conventional touch-based interfaces toward more natural, contactless, and context-aware interaction paradigms. Among these paradigms, dynamic hand gesture recognition has attracted substantial attention because it enables intuitive command delivery in smart environments, immersive interaction, and assistive applications. Existing gesture recognition systems are mainly built on wearable sensing or vision-based perception. Wearable systems can provide stable motion measurements, but they often introduce donning burden and reduce interaction convenience. Vision-based approaches offer rich appearance cues, yet their performance is still vulnerable to illumination variation, self-occlusion, and privacy concerns in practical scenarios. These limitations have motivated growing interest in radio-frequency (RF) sensing, which offers contactless operation and stronger robustness under challenging environmental conditions. In particular, ultra-wideband (UWB) radar is highly attractive because of its fine temporal resolution, privacy-preserving nature, and insensitivity to lighting conditions (Tchantchane et al., 2023; Huang et al., 2021; Khunteta et al., 2022; Ahmed et al., 2021a).

Among RF sensing technologies, impulse-radio ultra-wideband (IR-UWB) radar has become a promising modality for gesture recognition. Its large bandwidth enables the capture of subtle hand motion variations, while its non-visual sensing mechanism makes it naturally suitable for privacy-sensitive HCI scenarios. Early IR-UWB gesture studies mainly relied on handcrafted signal processing and conventional classifiers. Representative early studies investigated gesture recognition using IR-UWB radar sensors and further explored hand-based gesture recognition for vehicular applications (Ren et al., 2016; Khan et al., 2017). With the rapid development of deep learning, radar gesture recognition has gradually shifted from handcrafted representations toward data-driven feature learning. It has been shown that IR-UWB radar signatures can be effectively modeled by an inception-module-based classifier (Ahmed and Cho, 2020). Subsequently, a public 12-class benchmark, UWB-Gestures, was released to improve reproducibility and comparative evaluation in this field (Ahmed et al., 2021b).

Beyond IR-UWB-specific studies, the broader radar gesture recognition literature has explored a wide range of modeling paradigms. For FMCW and Doppler radar, prior studies investigated feature-based hand gesture recognition with temporal feature analysis and proposed dynamic continuous hand gesture recognition frameworks using FMCW radar (Ryu et al., 2018; Zhang et al., 2018). More recently, Transformer-based, lightweight, and multi-feature fusion architectures have been introduced to improve long-range temporal modeling, deployment efficiency, and feature discriminability in radar-based gesture recognition. A vision-transformer framework with a convolutional encoder–decoder has been developed for Doppler-radar hand gesture recognition (Kehelella et al., 2022). In parallel, recent studies have explored range–velocity–angle fusion, range–time/angle–time multifeature fusion, and multi-stream CNN designs to exploit complementary radar representations for more robust recognition (Yu et al., 2024; Wu et al., 2024; Park and Jeong, 2025). In addition, highly optimized lightweight radar gesture recognition networks and embedded radar models have been reported to improve deployment efficiency under resource-constrained conditions (Chmurski et al., 2021; Scherer et al., 2021). In parallel, several review papers have summarized the rapid progress of radar-based gesture sensing, including general radar-sensor-based hand gesture recognition, FMCW-radar hand gesture recognition, and dynamic gesture recognition based on FMCW millimeter-wave radar (Wang et al., 2022b; Tang et al., 2023). These studies demonstrate that radar gesture recognition has evolved into a mature and active research area, but they also reveal that robustness, generalization, and efficient spatiotemporal modeling remain open challenges.

To improve robustness, fusion-based perception has increasingly been explored. On the one hand, multimodal fusion has been shown to be effective for gesture recognition in complex environments. Radar–vision fusion has been introduced for multimodal dynamic hand gesture recognition, and cross-modal learning between camera-derived and radar point-cloud representations has also been investigated for long-range gesture recognition (Liu and Liu, 2023; Hazra et al., 2022). On the other hand, the importance of complementary observations is especially significant in radar sensing because radar signatures are highly viewpoint-dependent. The same gesture may produce substantially different responses when observed from different directions, and single-view sensing is thus inherently vulnerable to self-occlusion, orientation variation, and incomplete spatial exposure. Although the public UWB-Gestures dataset itself was collected using three impulse radars from different viewpoints, the effective exploitation of multi-view complementary information within an end-to-end radar recognition framework remains insufficiently studied.

Another relevant research direction is neuromorphic and event-driven computing. Neuromorphic systems emphasize sparse, event-sensitive, and energy-efficient spatiotemporal information processing, often inspired by biological neural computation (Indiveri and Liu, 2015; Neftci, 2018). In radar gesture recognition, several recent studies have started to investigate neuromorphic models more directly. For example, spiking neural networks have been introduced for IR-UWB radar hand gesture recognition, and resource-efficient gesture sensing systems based on mm-wave radar have also been developed under the neuromorphic computing paradigm (Wang et al., 2022a; Arsalan et al., 2022). However, most of these studies mainly focus on spiking implementations or edge efficiency. In contrast, the problem of multi-view radar gesture perception with jointly modeled local and global dynamics remains largely underexplored. This gap is particularly relevant for IR-UWB radar, where motion-enhanced time-range (TR) representations contain both fine-grained local texture variations and long-range temporal evolution.

Motivated by the above observations, we formulate this work as a neuromorphic-inspired multi-view radar perception framework. Here, “neuromorphic-inspired” refers to an algorithm-level design principle rather than a fully spiking neural network or a dedicated neuromorphic hardware implementation. In this study, the inspiration is mainly reflected in three aspects: motion-sensitive radar representation, global-local collaborative feature modeling, and adaptive feature selection. Specifically, motion-enhanced TR maps emphasize dynamic radar responses while suppressing static clutter; the parallel global-local branches jointly encode fine-grained motion details and long-range temporal context; and the adaptive fusion module selectively integrates complementary feature streams instead of using a fixed fusion rule.

Based on this motivation, we propose a neuromorphic-inspired multi-view global-local fusion network for IR-UWB radar dynamic gesture recognition, hereafter referred to as MV-GLFNet. Specifically, motion-enhanced TR maps from three complementary viewpoints are first integrated through early fusion to improve the spatial completeness of radar observations. A dual-branch architecture is then employed to encode local dynamic textures and global temporal structures in parallel, followed by an adaptive fusion module to enhance feature complementarity and representation discriminability. The main contributions of this work are summarized as follows:

1. We propose a neuromorphic-inspired multi-view IR-UWB radar gesture recognition framework that exploits complementary motion-enhanced TR maps from three viewpoints.
2. We design MV-GLFNet, a global-local dual-branch network, to jointly capture local dynamic textures and global temporal dependencies.
3. We introduce an adaptive fusion strategy that combines gated first-order fusion and bilinear second-order interaction for more effective feature integration.
4. We validate the proposed framework on a public 12-class UWB gesture dataset under a subject-independent protocol, achieving 98.29% average accuracy and outperforming several representative baselines.

## 2 Signal modeling and pre-processing

### 2.1 Signal model

For clarity, the signal model is first described for a single IR-UWB radar viewpoint. The same formulation applies independently to the Left, Top, and Right radars in the proposed multi-view acquisition setup. Since the subsequent recognition framework operates on TR representations, it is necessary to describe how gesture motion is encoded in the received echoes and organized into a two-dimensional radar data matrix.

IR-UWB radar transmits a sequence of ultra-short baseband pulses rather than a continuous narrowband carrier. Owing to its large instantaneous bandwidth, it provides high temporal resolution and fine range resolution, making it suitable for short-range motion sensing and dynamic gesture recognition. Let *p*(*t*) denote a single transmitted IR-UWB pulse, which is typically modeled as a Gaussian-derivative monocycle. Over one coherent processing interval, the transmitted pulse train *s*_tx_(*t*) is given by Equation 1:


$$\begin{array}{l} s_{\text{tx}}\left(t\right)=\overset{M-1}{\underset{m=0}{\sum }}p\left(t-mT_{f}\right) \end{array}$$ (1)

where *t* denotes continuous time, *m* is the pulse index, *M* is the total number of transmitted pulses within one coherent processing interval, and *T*_*f*_ is the pulse repetition interval. If the signal bandwidth is *B*, the theoretical range resolution is $\Delta R=\frac{c}{2B}$.The large bandwidth therefore enables the radar to resolve small spatial displacements caused by hand and finger motion.

To describe the motion-dependent echo formation, a dominant target-path model is first considered. Let *R*_0_ denote the initial target range, and let Δ*R*(*t*) denote the time-varying range displacement induced by target motion. Then, at the *m*-th transmitted pulse, the instantaneous target range *R*(*m*) is given by Equation 2:


$$\begin{array}{l} R\left(m\right)=R_{0}+\Delta R\left(mT_{f}\right) \end{array}$$ (2)

and the corresponding round-trip propagation delay τ_*m*_ is given by Equation 3:


$$\begin{array}{l} \tau_{m}=\frac{2R\left(m\right)}{c}=\frac{2\left(R_{0}+\Delta R\left(mT_{f}\right)\right)}{c} \end{array}$$ (3)

thus, gesture motion is directly reflected as a time-varying delay in the received echo.

Based on the above range–delay relationship, the received echo from the dominant moving target, *s*_rx_(*t*), is given by Equation 4:


$$\begin{array}{l} s_{\text{rx}}\left(t\right)=\overset{M-1}{\underset{m=0}{\sum }}A_{m}p\left(t-mT_{f}-\tau_{m}\right)+s_{\text{cl}}\left(t\right)+n\left(t\right) \end{array}$$ (4)

where *A*_*m*_ denotes the amplitude coefficient of the target echo at the *m*-th pulse, *s*_cl_(*t*) represents static background clutter and environmental reflections, and *n*(*t*) denotes additive noise. The motion information is mainly encoded in the delay variation τ_*m*_ and the amplitude variation *A*_*m*_.

In practical indoor environments, the received signal with multiple propagation paths, *r*(*t*), is given by Equation 5:


$$\begin{array}{l} r\left(t\right)=\overset{L}{\underset{i=1}{\sum }}\alpha_{i}\left(t\right)p\left(t-\tau_{i}\left(t\right)\right)+n\left(t\right) \end{array}$$ (5)

where *L* is the number of effective propagation paths, and α_*i*_(*t*) and τ_*i*_(*t*) are the time-varying amplitude and delay of the *i*-th path, respectively. In this form, target echoes, environmental reflections, and multipath components are all incorporated into the received signal.

During acquisition, the received echo is repeatedly sampled within each pulse repetition interval. Samples within a single pulse interval describe the echo distribution along the delay axis and correspond to the range dimension, referred to as fast time. The sequence of echoes across successive transmitted pulses characterizes the temporal evolution of the target motion and forms the slow-time dimension. By arranging the measurements in this way, the received signal can be reorganized into a two-dimensional fast-time–slow-time representation. Let *t*_*f*_ denote the fast-time variable within one pulse repetition interval. Then, for the *m*-th pulse, the received echo *y*(*t*_*f*_, *m*) is given by Equation 6:


$$\begin{array}{l} y\left(t_{f},m\right)=A_{m}p\left(t_{f}-\tau_{m}\right)+c\left(t_{f},m\right)+n\left(t_{f},m\right) \end{array}$$ (6)

where *m* is the slow-time index, *c*(*t*_*f*_, *m*) denotes clutter and background components in the two-dimensional domain, and *n*(*t*_*f*_, *m*) is additive noise. Here, the fast-time axis represents the range-dependent echo profile, while the slow-time axis characterizes the temporal evolution of target motion.

After discrete sampling, the radar measurement *r*[*n, k*] is given by Equation 7:


$$\begin{array}{l} r\left[n,k\right]=r\left(nT_{f}+kT_{s}\right) \end{array}$$ (7)

where *n* = 0, 1, …, *N*−1 is the slow-time pulse index, *k* = 0, 1, …, *K*−1 is the fast-time sampling index, and *T*_*s*_ is the fast-time sampling interval. In this matrix, each row corresponds to a range profile at a specific slow-time instant, whereas each column describes the temporal variation of a particular range bin across successive pulses. The fast-time sample index k can be approximately mapped to the physical range *R*_*k*_ using Equation 8:


$$\begin{array}{l} R_{k}=\frac{ckT_{s}}{2} \end{array}$$ (8)

Therefore, the fast-time–slow-time matrix provides a compact two-dimensional spatiotemporal representation of gesture echoes and serves as the basis for the subsequent construction of TR maps.

### 2.2 Time–range map generation

Based on the fast-time–slow-time matrix in (8), a fixed-length temporal segment is extracted for each gesture instance and reorganized into a TR map. Specifically, consecutive range profiles are stacked along the slow-time dimension to form the two-dimensional TR representation TR(*n, k*), as defined in Equation 9:


$$\begin{array}{l} \text{TR}\left(n,k\right)=r\left[n,k\right] \end{array}$$ (9)

where *n* = 0, 1, …, *N*−1 denotes the slow-time index and *k* = 0, 1, …, *K*−1 denotes the fast-time index. In this representation, the horizontal axis corresponds to the temporal evolution of the gesture, whereas the vertical axis corresponds to the range dimension.

**Figure 1:**
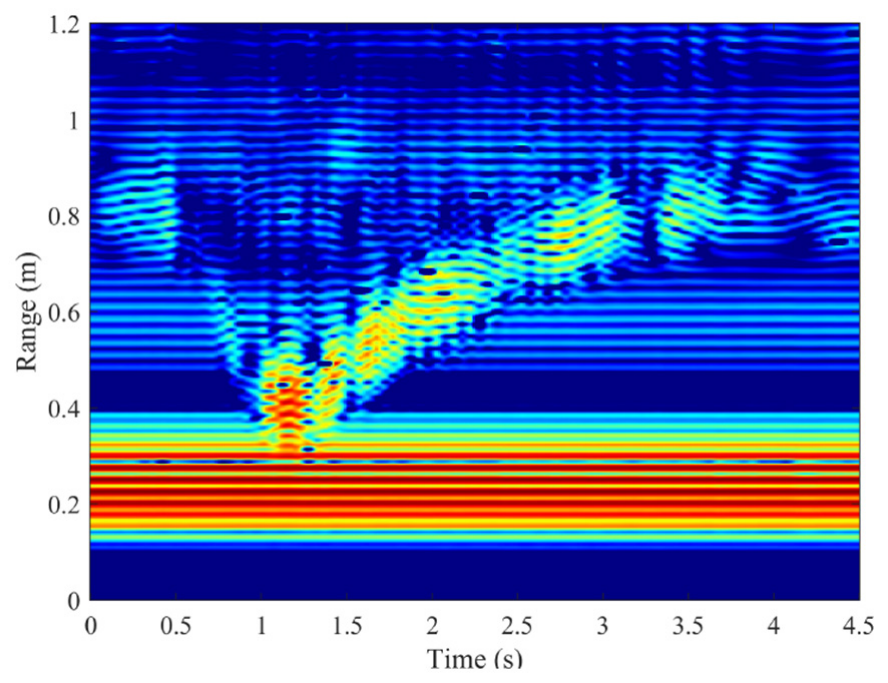
Raw time-range (TR) map before clutter suppression.

Compared with the original one-dimensional echo waveform, the TR representation provides a more structured description of gesture motion by jointly preserving spatial displacement and temporal evolution. Different dynamic gestures produce distinct energy trajectories in the TR domain due to differences in motion direction, amplitude, and temporal pattern. The TR map is therefore well-suited for subsequent feature extraction and classification. An example of the raw TR representation is shown in Figure 1. It can be observed that the gesture-induced energy is distributed in the TR plane, while strong background components are also present.

However, the raw TR map usually contains strong static or quasi-static components caused by background reflectors, such as walls, tables, and the radar body itself. These components remain nearly unchanged over adjacent slow-time instants and may obscure the weaker motion-induced signatures. To suppress such background interference, a moving target indicator (MTI) filter (Ash et al., 2018) is applied along the slow-time dimension. The MTI-filtered TR map TR_MTI_(*n, k*) is defined by Equation 10:


$$\begin{array}{l} {\text{TR}}_{\text{MTI}}\left(n,k\right)=\text{TR}\left(n,k\right)-\text{TR}\left(n-1,k\right) \end{array}$$ (10)

This operation suppresses stationary clutter and enhances motion-induced variations in the TR map. As a temporal high-pass operation along the slow-time dimension, first-order differential MTI may attenuate slow-moving or low-amplitude gesture responses. Therefore, its benefit in clutter suppression is accompanied by a practical trade-off for gestures with weak temporal variations.

After MTI filtering, each radar viewpoint yields a motion-enhanced TR map. Since the proposed system employs three IR-UWB radars placed at different viewpoints, the above procedure is performed independently for the Left, Top, and Right views. The resulting three-view TR maps are then used as the input representations of the subsequent recognition framework. In this way, both the motion characteristics within each view and the complementary information across multiple viewpoints can be effectively exploited.

## 3 Method

### 3.1 Overall framework

**Figure 2:**
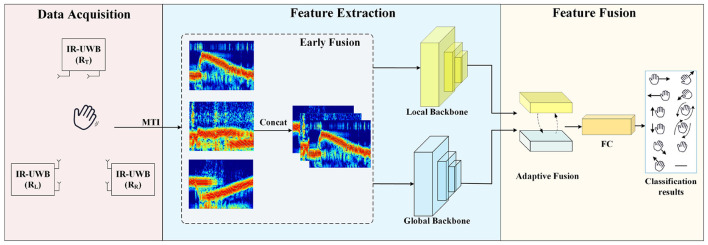
Overall framework of the proposed MV-GLFNet for multi-view IR-UWB radar gesture recognition.

The overall architecture of the proposed MV-GLFNet is illustrated in Figure 2, which comprises three stages: data acquisition, feature extraction, and feature fusion. In the data acquisition stage, three IR-UWB radars collect gesture echoes from different viewpoints. After MTI filtering, a TR map is generated for each view. Since the three TR maps are generated from the same IR-UWB modality and share aligned TR dimensions after identical preprocessing, they can be naturally organized as multi-channel inputs. This early fusion design allows cross-view motion cues to be jointly encoded from the input stage, thereby reducing viewpoint-dependent information loss. Compared with intermediate or late fusion, it avoids additional view-specific backbones and provides a compact yet effective solution for homogeneous multi-view TR representations.

In the feature extraction stage, the fused input is processed by two parallel branches, namely the Local Backbone and the Global Backbone. The Local Backbone is designed to capture fine-grained spatial textures and short-term dynamic patterns, whereas the Global Backbone models long-range dependencies and the overall temporal evolution in the TR domain. The extracted local and global features are then integrated by the Adaptive Fusion module to obtain a more informative and discriminative fused representation.

Finally, the fused feature vector is projected into the class space through a fully connected (FC) layer, followed by a Softmax function to generate gesture probabilities. The class with the highest probability is selected as the final recognition result, thereby completing the end-to-end recognition process. The key techniques of the feature extraction and fusion modules are detailed in the following subsections.

### 3.2 Local backbone

**Figure 3:**
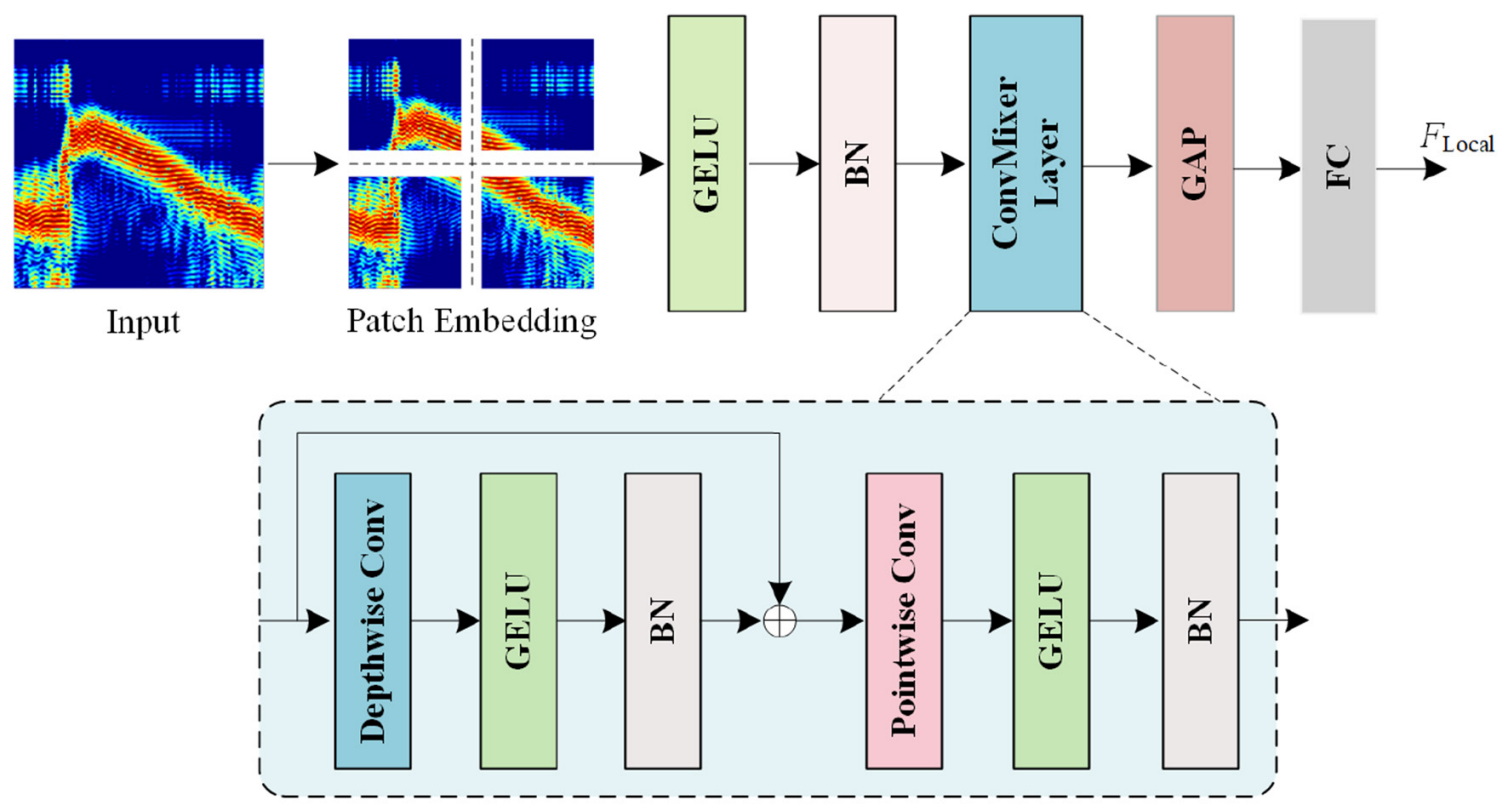
Architecture of the local backbone for extracting fine-grained local TR features.

To capture fine-grained local dynamic patterns in the TR maps that are critical for gesture classification, we design a Local Backbone based on ConvMixer (Trockman and Kolter, 2022), as illustrated in Figure 3. This branch aims to preserve spatial structure while enhancing local pattern representation, thereby effectively modeling the short-term, localized, and directional energy variations inherent in UWB radar gestures. Compared with conventional hierarchical CNNs, the isotropic architecture of ConvMixer alleviates the spatial resolution loss caused by aggressive downsampling, which is beneficial for preserving subtle local details in TR representations. In addition, by alternating depthwise and pointwise convolutions (Chollet, 2017), ConvMixer enables efficient spatial and channel mixing while maintaining a strong local inductive bias.

Given an input TR representation *X*∈ℝ^3 × 64 × 128^, a convolutional patch embedding layer is first applied to partition the input into non-overlapping 2 × 2 patches and project it into a higher-dimensional feature space. This patch embedding operation can be implemented as a 2D convolution with kernel size 2 and stride 2, as given in Equation 11:


$$\begin{array}{l} Z\left[c^{\prime },i,j\right]=\overset{C_{\text{in}}-1}{\underset{c=0}{\sum }}\overset{1}{\underset{p=0}{\sum }}\overset{1}{\underset{q=0}{\sum }}X\left[c,2i+p,2j+q\right]W_{\text{e}}\left[c^{\prime },c,p,q\right]+b_{\text{e}}\left[c^{\prime }\right] \end{array}$$ (11)

where *W*_e_ and *b*_e_ denote the weights and biases of the patch embedding layer, respectively, and *c*′ indexes the output channels. After patch embedding, the spatial resolution is reduced from 64 × 128 to 32 × 64, while the channel dimension is increased from 3 to 256. The embedded features are then processed by GELU activation and batch normalization (BN) to enhance non-linearity and stabilize training.

After obtaining the initial feature representation, stacked ConvMixer layers are used to further strengthen local spatial modeling and cross-channel interaction. Each ConvMixer layer consists of a depthwise convolution, a residual connection, and a pointwise convolution, followed by GELU activation and BN. For an input feature map *X*∈ℝ^*C*×*H*×*W*^, the depthwise convolution operation *X*_d_ is given by Equation 12:


$$\begin{array}{l} X_{\text{d}}\left[c,i,j\right]=\overset{K-1}{\underset{p=0}{\sum }}\overset{K-1}{\underset{q=0}{\sum }}X\left[c,i+p,j+q\right]W_{\text{d}}\left[c,p,q\right] \end{array}$$ (12)

where $W_{\text{d}}\in {\mathbb{R}}^{C\times K\times K}$ denotes the depthwise convolution kernel and *K* is the kernel size. To preserve the original representation while incorporating local spatial cues, a residual connection is introduced, as given in Equation 13:


$$\begin{array}{l} X_{\text{r}}=X+\text{BN}\left(\text{GELU}\left(X_{\text{d}}\right)\right) \end{array}$$ (13)

Subsequently, a pointwise convolution is applied to fuse information across channels while preserving the spatial resolution, as given in Equation 14:


$$\begin{array}{l} X_{\text{p}}\left[c^{\prime },i,j\right]=\overset{C-1}{\underset{c=0}{\sum }}X_{\text{r}}\left[c,i,j\right]W_{\text{p}}\left[c^{\prime },c\right] \end{array}$$ (14)

where *W*_p_ denotes the 1 × 1 pointwise convolution kernel, and *C* and *C*′ are the input and output channel numbers, respectively. The pointwise convolution is followed by GELU activation and BN to improve feature expressiveness and optimization stability.

To progressively enhance local feature representation, multiple ConvMixer layers are stacked. Considering the trade-off between computational complexity and recognition performance, we employ *n* = 4 ConvMixer layers in the Local Backbone. After the stacked layers, global average pooling (GAP) is used to aggregate spatial information into a channel-wise descriptor, as given in Equation 15:


$$\begin{array}{l} y\left[c\right]=\frac{1}{HW}\overset{H-1}{\underset{i=0}{\sum }}\overset{W-1}{\underset{j=0}{\sum }}X_{\text{p}}\left[c,i,j\right] \end{array}$$ (15)

Finally, a fully connected (FC) layer projects the pooled descriptor into the target feature space for subsequent fusion with the global branch, as given in Equation 16:


$$\begin{array}{l} F_{\text{Local}}=W_{\text{fc}}y+b_{\text{fc}} \end{array}$$ (16)

where *W*_fc_ and *b*_fc_ are the weights and biases of the FC layer, and *F*_Local_ denotes the final local feature representation.

### 3.3 Global backbone

**Figure 4:**
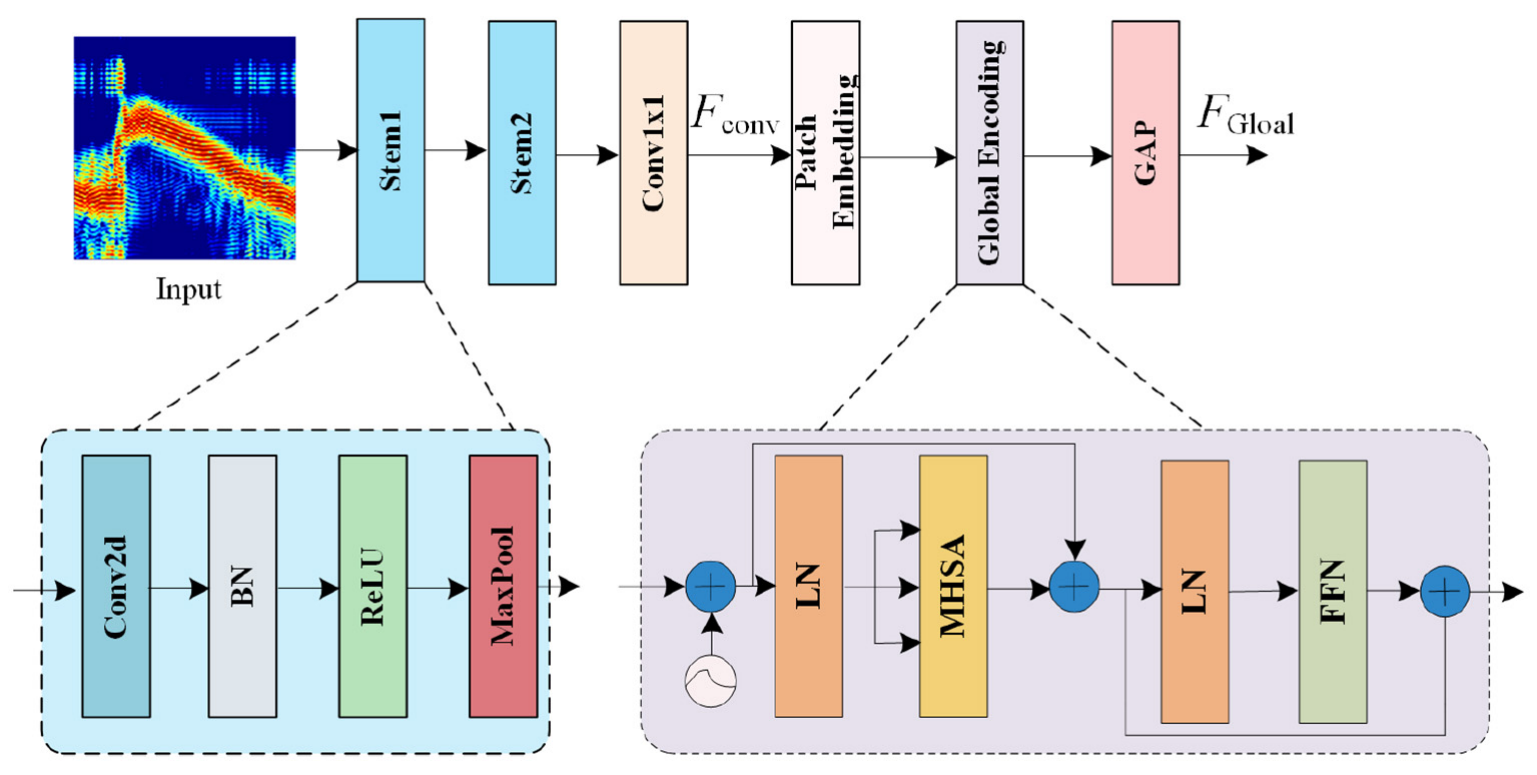
Architecture of the global backbone for modeling long-range temporal dependencies.

To capture global spatiotemporal dependencies in the TR maps that are critical for gesture recognition, we design a Global Backbone based on a lightweight CNN–Transformer architecture, as illustrated in Figure 4. Unlike the Local Backbone, which focuses on fine-grained local dynamic variations, the Global Backbone is intended to model long-range interactions, global temporal evolution, and overall energy distribution patterns in the TR domain, thereby providing high-level semantic representations complementary to the local features.

Given an input TR representation *X* of size 3 × 64 × 128, a lightweight two-stage convolutional stem is first employed to extract low-level features and reduce the spatial resolution. Each stage consists of a convolution layer, batch normalization (BN), ReLU activation, and a 2 × 2 max-pooling layer. After the two stages, the feature map resolution is reduced to 16 × 32, and the channel dimension is increased to 64. To project the extracted feature into the Transformer embedding space, a 1 × 1 convolution is further applied, yielding Equation 17:


$$\begin{array}{l} F_{\text{conv}}={\text{Conv}}_{1\times 1}\left(\text{Stem}\left(X\right)\right) \end{array}$$ (17)

where *F*_conv_ is the projected feature map of size 256 × 16 × 32.

After obtaining the intermediate feature representation, a patch embedding layer is introduced to convert the 2D feature map into a token sequence suitable for Transformer-based global modeling. Specifically, a convolution with kernel size 2 and stride 2 is applied to partition *F*_conv_ into non-overlapping patches, as given in Equation 18:


$$\begin{array}{l} Z=\text{PatchEmbed}\left(F_{\text{conv}}\right) \end{array}$$ (18)

where *Z*∈ℝ^*N*×*d*^ denotes the token sequence, *N* = 8 × 16 = 128 is the number of tokens, and *d* = 256 is the embedding dimension. A layer normalization is then applied to the token sequence to stabilize the subsequent Transformer encoding (Vaswani et al., 2017).

To preserve the spatial ordering information of the tokenized TR representation, a learnable positional embedding is added before the Transformer encoder, as given in Equation 19:


$$\begin{array}{l} Z^{\left(0\right)}=Z+E_{\text{pos}} \end{array}$$ (19)

where $E_{\text{pos}}\in {\mathbb{R}}^{N\times d}$ denotes the learnable positional embedding. A dropout layer is further applied to *Z*^(0)^ for regularization.

After patch embedding and positional encoding, the token sequence is fed into stacked Transformer blocks to model long-range dependencies and global contextual relationships. Each Transformer block adopts a pre-normalization structure with multi-head self-attention (MHSA) and a feed-forward network (FFN). For the input sequence *Z*^(*l*−1)^ of the *l*-th block, the query, key, and value matrices are first obtained through linear projections, as given in Equation 20:


$$\begin{array}{l} Q=Z^{\left(l-1\right)}W_{Q},\;K=Z^{\left(l-1\right)}W_{K},\;V=Z^{\left(l-1\right)}W_{V} \end{array}$$ (20)

where *W*_*Q*_, *W*_*K*_, and *W*_*V*_ are learnable projection matrices. The scaled dot-product attention for each head is formulated as Equation 21:


$$\begin{array}{l} \text{Attention}\left(Q,K,V\right)=\text{softmax}\left(\frac{QK^{⊤}}{\sqrt{d_{k}}}\right)V \end{array}$$ (21)

where *d*_*k*_ denotes the feature dimension of each attention head. By explicitly modeling pairwise correlations among all tokens, MHSA enables the network to capture long-range interactions and global temporal structures in the TR domain.

To preserve the original representation while incorporating global contextual information, residual connections are employed around both the attention branch and the FFN branch. The *l*-th Transformer block can therefore be written as Equations 22 and 23:


$$\begin{array}{l} {\tilde{Z}}^{\left(l\right)}=Z^{\left(l-1\right)}+\text{MHSA}\left({\text{LN}}_{1}\left(Z^{\left(l-1\right)}\right)\right) \end{array}$$ (22)

$$\begin{array}{l} Z^{\left(l\right)}={\tilde{Z}}^{\left(l\right)}+\text{MLP}\left({\text{LN}}_{2}\left({\tilde{Z}}^{\left(l\right)}\right)\right) \end{array}$$ (23)

where LN_1_(·) and LN_2_(·) denote layer normalization, and MLP(·) represents the feed-forward network with GELU activation. In our implementation, the Transformer branch uses an embedding dimension of *d* = 256, with 8 attention heads and *L* = 4 stacked encoder blocks. The FFN hidden dimension is set to 1, 024.

After the stacked Transformer blocks, a final layer normalization is applied to the output token sequence, as given in Equation 24:


$$\begin{array}{l} Z_{\text{out}}=\text{LN}\left(Z^{\left(L\right)}\right) \end{array}$$ (24)

To obtain a compact global descriptor, average pooling is performed over the token dimension, as given in Equation 25:


$$\begin{array}{l} F_{\text{Global}}=\frac{1}{N}\overset{N}{\underset{n=1}{\sum }}Z_{\text{out}}\left[n\right]. \end{array}$$ (25)

This operation plays the role of global average pooling over the tokenized representation and yields the final global feature vector $F_{\text{Global}}\in {\mathbb{R}}^{d}$. The resulting feature summarizes the long-range spatiotemporal dependencies of the input TR representation and is subsequently used in the adaptive fusion stage.

### 3.4 Adaptive feature fusion

**Figure 5:**
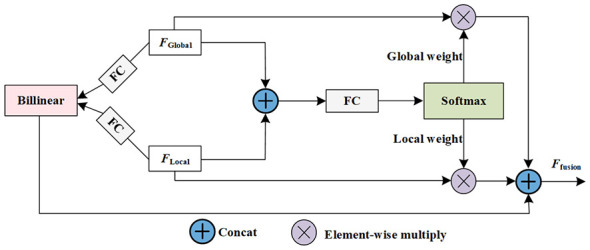
Adaptive fusion module for integrating local and global feature representations.

After the Local Backbone and Global Backbone extract complementary local and global features, the resulting feature vectors are fed into the Adaptive Feature Fusion module, as illustrated in Figure 5. Compared with fixed fusion operations, the proposed adaptive fusion strategy provides a more flexible way to integrate local and global TR representations. Concatenation does not explicitly model feature complementarity, addition relies on a rigid linear combination, and multiplication may suppress useful cues when one branch response is weak. By combining gated first-order fusion with bilinear second-order interaction, the proposed module adaptively balances the two branches and explicitly captures cross-branch correlations, thereby enhancing the discriminability of the fused representation.

Let $F_{\text{Local}}\in {\mathbb{R}}^{D}$ and $F_{\text{Global}}\in {\mathbb{R}}^{D}$ denote the local and global features, respectively. Since the local and global branches provide complementary TR representations, the fusion module is designed to capture both adaptive branch-level importance and cross-branch feature interactions. Specifically, the first-order gated branch dynamically weights the local and global features, while the second-order bilinear branch models their multiplicative correlations. This complementary design strengthens the discriminability of the fused representation. To model second-order multiplicative interactions between the two branches, a bilinear fusion strategy is adopted. To reduce the parameter complexity, both features are first projected into a lower-dimensional latent space, as given in Equation 26:


$$\begin{array}{l} {\tilde{F}}_{\text{Local}}=F_{\text{Local}}W_{\text{l}}+b_{\text{l}},\;{\tilde{F}}_{\text{Global}}=F_{\text{Global}}W_{\text{g}}+b_{\text{g}} \end{array}$$ (26)

where *W*_l_ and *W*_g_ are learnable projection matrices, and *b*_l_ and *b*_g_ are the corresponding biases. In our implementation, both features are projected to a 128-dimensional latent space. The projected features are then combined through a bilinear operator (Lin et al., 2015) to obtain the second-order interaction feature, as given in Equation 27:


$$\begin{array}{l} F_{\text{bi}}=B\left({\tilde{F}}_{\text{Local}},{\tilde{F}}_{\text{Global}}\right) \end{array}$$ (27)

where B(·,·) denotes the bilinear transformation. This branch explicitly captures cross-feature correlations that cannot be modeled by linear fusion alone.

In addition to second-order interaction modeling, an adaptive gating branch is introduced to dynamically balance the contributions of local and global features. Specifically, the two feature vectors are first concatenated and then fed into a lightweight gating network to generate the gating score G, as given in Equation 28. The adaptive fusion weights are then obtained through Softmax, as given in Equation 29:


$$\begin{array}{l} G=\text{MLP}\left(\text{Concat}\left(F_{\text{Local}},F_{\text{Global}}\right)\right) \end{array}$$ (28)

$$\begin{array}{l} \left[w_{\text{Local}},w_{\text{Global}}\right]=\text{Softmax}\left(G\right) \end{array}$$ (29)

where *w*_Local_ and *w*_Global_ denote the adaptive weights assigned to the local and global branches, respectively. Based on these weights, the first-order fused feature is obtained as given in Equation 30:


$$\begin{array}{l} F_{\text{fo}}=w_{\text{Local}}F_{\text{Local}}+w_{\text{Global}}F_{\text{Global}}. \end{array}$$ (30)

This weighted combination enables the network to adaptively emphasize the more informative branch for different gesture instances.

Finally, the first-order gated feature and the second-order bilinear feature are concatenated to form the final hybrid representation, as given in Equation 31:


$$\begin{array}{l} F_{\text{fusion}}=\text{Concat}\left(F_{\text{fo}},F_{\text{bi}}\right) \end{array}$$ (31)

By jointly preserving first-order complementary information and second-order interaction cues, the fused feature *F*_fusion_ provides a richer representation for subsequent gesture classification.

The fused feature *F*_fusion_ is fed into a fully connected classifier for gesture prediction. The network is trained using the cross-entropy loss, as given in Equation 32:


$$\begin{array}{l} L=-\overset{C}{\underset{i=1}{\sum }}y_{i}\log{ŷ}_{i} \end{array}$$ (32)

where *C* is the number of gesture classes, *y*_*i*_ is the one-hot encoded ground-truth label, and ŷ_*i*_ is the predicted probability of the *i*-th class.

## 4 Experiments and results

### 4.1 Dataset

**Figure 6:**
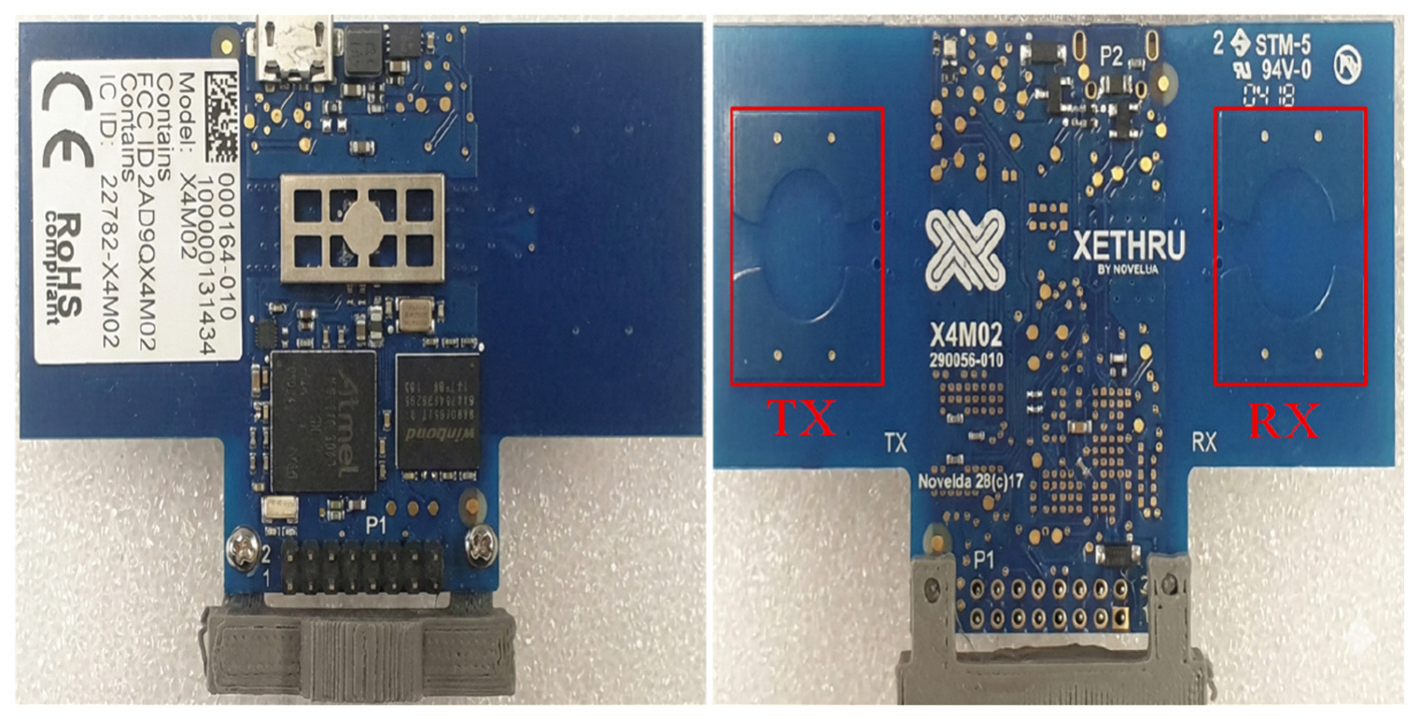
IR-UWB radar system.

The proposed method is evaluated on a public UWB gesture recognition dataset (Ahmed et al., 2021b). Data acquisition is conducted using the XeThru X4 pulse-based IR-UWB radar developed by Novelda, which offers low power consumption and high integration and is therefore suitable for near-field sensing applications, as shown in Figure 6. The main radar parameters are listed in Table 1. In particular, the radar operates at a center frequency of 8.745 GHz with a bandwidth of 1.5 GHz and a pulse repetition frequency of 40.5 MHz. A transmit–receive antenna pair is employed for pulse transmission and echo reception, and the radar data are collected at 20 fps.

**Table 1:** Parameters of the IR-UWB radar system.

Parameter	Value
Center frequency	8.745 GHz
Signal bandwidth	1.5 GHz
Frame rate	20 frames/s
Pulse repetition frequency	40.5 MHz
Antenna configuration	1 Tx and 1 Rx

**Figure 7:**
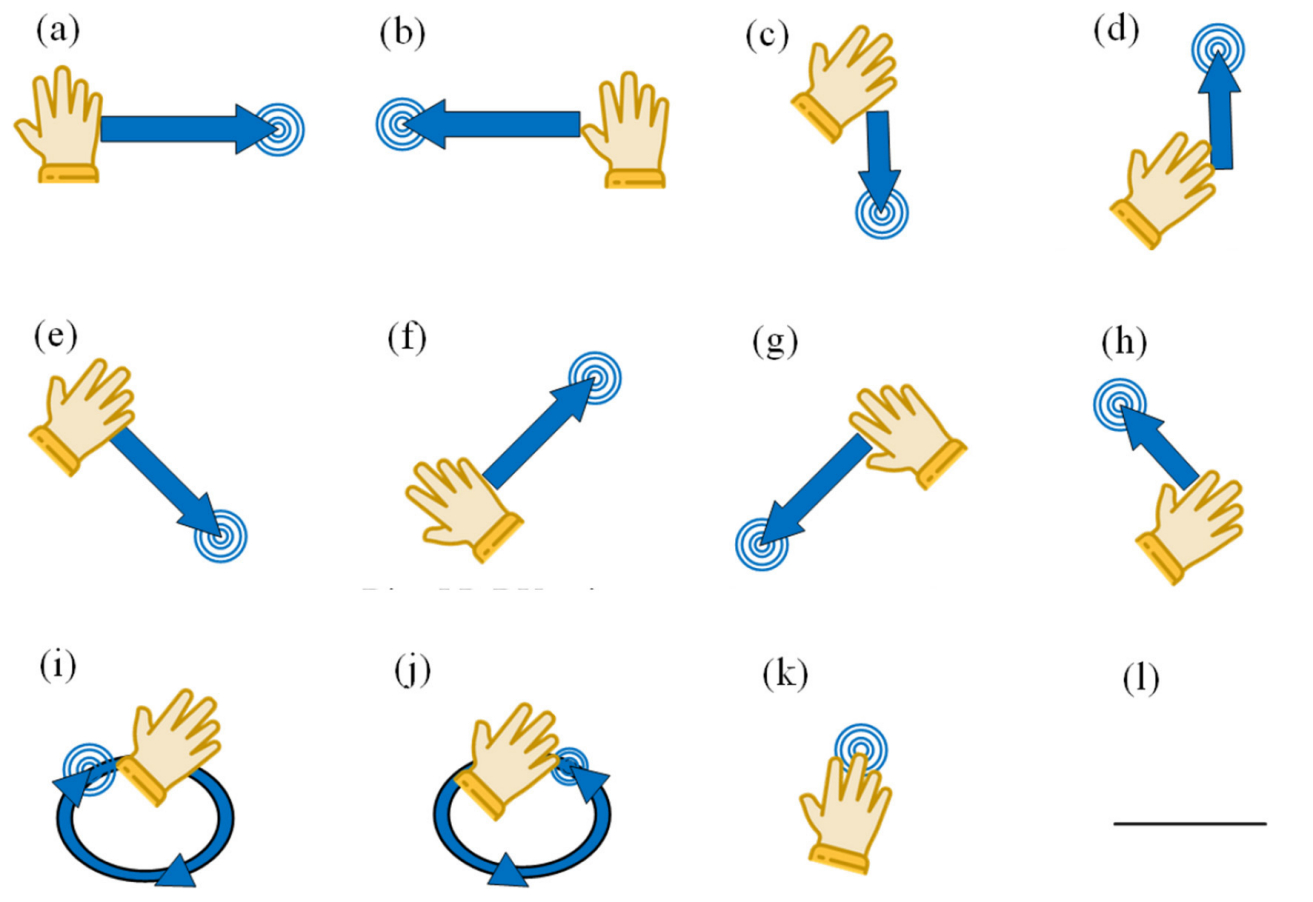
Hand gestures. **(a)** Left-right swipe, **(b)** right-left swipe, **(c)** Up down swipe, **(d)** Down up swipe, **(e)** Diag-LR-UD swipe, **(f)** Diag-LR-DU swipe, **(g)** Diag-RL-UD swipe, **(h)** Diag-RL-DU swipe, **(i)** clockwise, **(j)** anti-clockwise, **(k)** inward push, **(l)** empty.

The dataset consists of 12 gesture categories, as shown in Figure 7, including eight directional swipes, two rotations, one inward push, and one no-gesture category corresponding to the resting state. Specifically, the directional gestures include left–right, right–left, top–bottom, bottom–top, top-left to bottom-right, bottom-left to top-right, top-right to bottom-left, and bottom-right to top-left, while the rotational gestures include clockwise and counterclockwise motions. Data acquisition is performed using three pulse-based UWB radars (RL, RT, and RR) placed on the left, top, and right sides of the experimental platform, respectively. The gesture interaction region is located in the central area enclosed by the three radars, with a horizontal spacing of 1.1 m between RL and RR and a vertical distance of 0.55 m from RT to the midpoint of the horizontal pair.

**Figure 8:**
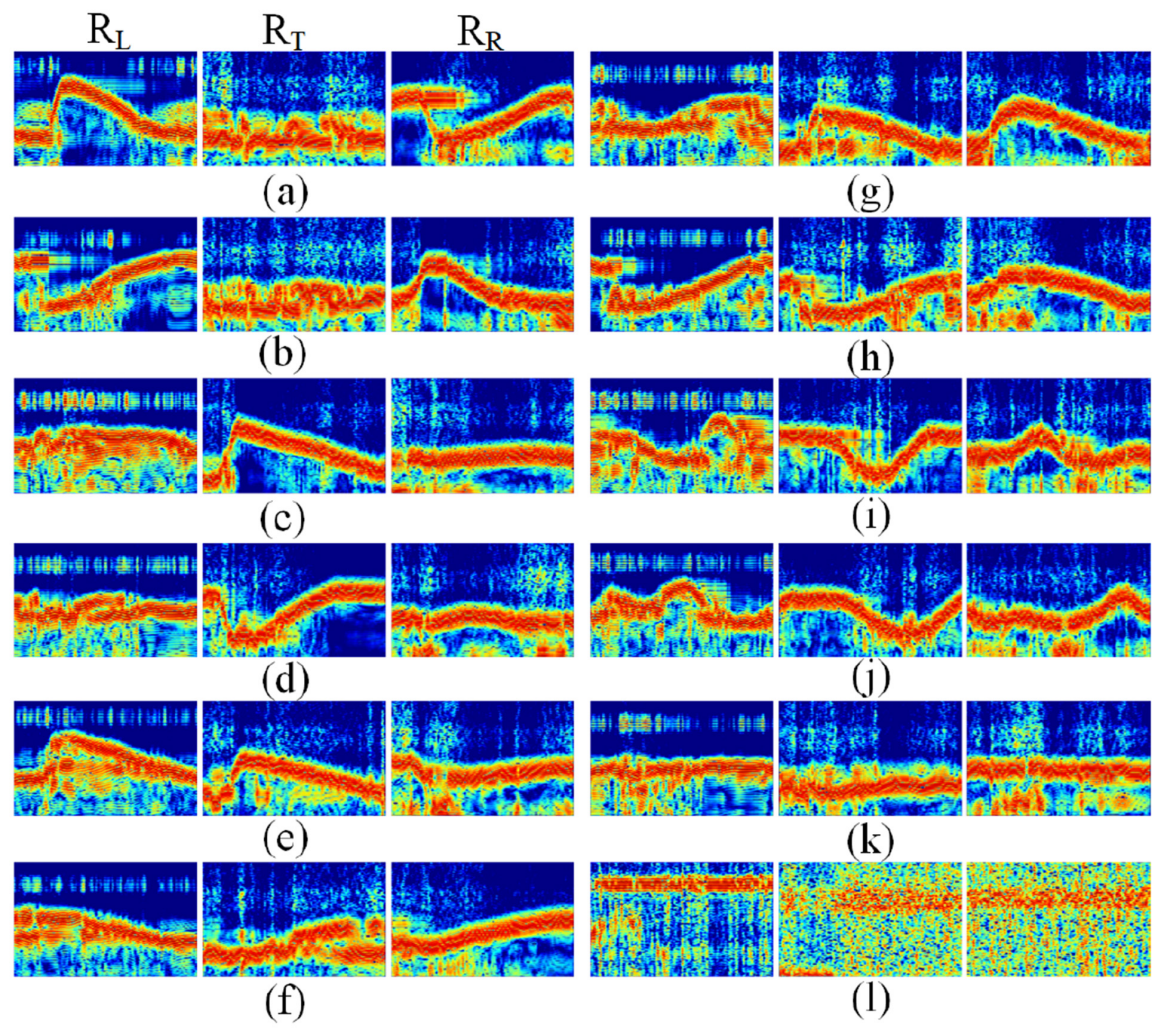
TR maps of all gestures from three views. **(a)** Left-right swipe, **(b)** right-left swipe, **(c)** up-down swipe, **(d)** down-up swipe, **(e)** Diag-LR-UD swipe, **(f)** Diag-LR-DU swipe, **(g)** Diag-RL-UD swipe, **(h)** Diag-RL-DU swipe, **(i)** clockwise, **(j)** anti-clockwise, **(k)** inward push, and **(l)** empty.

After acquisition, the radar echoes from each view are processed using MTI filtering to suppress static background clutter and enhance motion-induced gesture responses, resulting in TR representations of size 90 × 189. The TR maps of all gesture categories from the three radar views are shown in Figure 8. To ensure a consistent input size for network training, each MTI-processed TR map is resized to 64 × 128 by bilinear interpolation along the slow-time and range dimensions, without any cropping operation. The same preprocessing procedure is applied to the Left, Top, and Right views before multi-view fusion.

The constructed dataset comprises 9,600 samples collected from 8 subjects. To objectively assess cross-subject generalization, a subject-independent protocol is adopted, where samples from 6 subjects (7,200 samples) are used for training and those from the remaining 2 subjects (2,400 samples) are used for testing. This setting ensures that no subject overlap exists between the training and testing sets.

### 4.2 Experimental parameter settings

The gesture recognition network is implemented using PyTorch. Training and evaluation are performed on a system running Ubuntu 22.04 with 128 GB RAM, an Intel Core i9-14900K CPU, and an NVIDIA RTX 4090 GPU. For training, we use the AdamW optimizer with an initial learning rate of 0.001 and a weight decay of 0.0005. The network is trained end-to-end using the cross-entropy loss, with a batch size of 32 and a total of 100 training epochs.

### 4.3 Experimental analysis

The performance of the proposed method is evaluated using four widely adopted classification metrics, namely Accuracy, Precision, Recall, and F1-score (Hossin and Sulaiman, 2015), which are defined in Equations 33–36:


$$\begin{array}{c} \text{Accuracy}=\frac{TP+TN}{TP+TN+FP+FN} \end{array}$$ (33)

$$\begin{array}{c} \text{Precision}=\frac{TP}{TP+FP} \end{array}$$ (34)

$$\begin{array}{c} \text{Recall}=\frac{TP}{TP+FN} \end{array}$$ (35)

$$\begin{array}{c} \text{F1-score}=2\cdot \frac{\text{Precision}\cdot \text{Recall}}{\text{Precision}+\text{Recall}} \end{array}$$ (36)

where *TP*, *TN*, *FP*, and *FN* denote the numbers of true positive, true negative, false positive, and false negative predictions, respectively. For the multi-class setting, Precision, Recall, and F1-score are computed on a per-class basis and then averaged across all classes. These metrics jointly provide a comprehensive evaluation of the recognition performance from the perspectives of overall accuracy and class-level discriminability.

#### 4.3.1 Local backbone experiment

To evaluate the feature extraction capability of the Local Backbone, several representative CNN architectures are selected as baselines, including ResNet18 (He et al., 2016), MobileNetV2 (Sandler et al., 2018), ShuffleNetV2 (Ma et al., 2018), and EfficientNetV2 (Tan and Le, 2021). The experimental results are summarized in Table 2.

**Table 2:** Comparison of the local backbone with representative CNN models.

Model	Accuracy (%)	Precision (%)	Recall (%)	F1-score (%)
ResNet18	95.54	95.74	95.54	95.51
MobileNetV2	94.75	94.91	94.75	94.70
ShuffleNetV2	93.17	93.45	93.17	93.07
EfficientNetV2	96.20	96.30	96.20	96.07
Local backbone	**96.96**	**97.18**	**96.96**	**96.12**

Bold values indicate the results of the proposed method or proposed component.

As shown in Table 2, the proposed Local Backbone achieves the best performance across all evaluation metrics among the compared CNN models. Specifically, it reaches an accuracy of 96.96%, a precision of 97.18%, a recall of 96.96%, and an F1-score of 96.12%. These results demonstrate the effectiveness of the proposed Local Backbone in extracting discriminative local features for gesture recognition. In particular, the ConvMixer-based design helps strengthen local representation learning by combining patch-wise feature mixing with convolutional operations, which is beneficial for capturing subtle spatial variations in the input feature maps.

#### 4.3.2 Global backbone experiment

In this section, the proposed Global Backbone is compared with several representative sequence modeling architectures, including LSTM (Hochreiter and Schmidhuber, 1997), BiLSTM (Schuster and Paliwal, 1997), GRU, and BiGRU, in terms of recognition performance. To ensure fairness, all compared methods are evaluated using the same data preprocessing pipeline, training settings, and evaluation metrics. The quantitative results are presented in Table 3.

**Table 3:** Comparison of the global backbone with representative sequential models.

Model	Accuracy (%)	Precision (%)	Recall (%)	F1-score (%)
CNN+LSTM	93.10	92.90	93.10	93.23
CNN+BiLSTM	93.50	93.26	93.50	93.46
CNN+GRU	93.36	93.56	93.36	93.26
CNN+BiGRU	94.28	94.59	94.28	94.17
Global backbone	**97.00**	**97.11**	**97.00**	**96.99**

Bold values indicate the results of the proposed method or proposed component.

The experimental results show that different RNN-based combinations achieve broadly similar performance, while bidirectional variants provide modest improvements over their unidirectional counterparts. For example, CNN+BiGRU attains an accuracy of 94.28%, which is slightly higher than those of the other RNN-based baselines. In contrast, the proposed Global Backbone achieves the best results across all evaluation metrics, with an accuracy of 97.00%. This improvement suggests that, compared with conventional recurrent architectures, the proposed design is more effective at modeling long-range temporal dependencies and capturing global contextual relationships across the entire sequence. Such a global modeling capability is particularly beneficial for action recognition, where discriminative motion patterns may span multiple time steps rather than being confined to local temporal transitions.

#### 4.3.3 Fusion strategy experiments

This section compares different feature fusion strategies to assess the effectiveness of the dual-branch architecture and their impact on recognition performance. Because the global and local branches encode complementary information, the fusion operation plays an important role in determining how effectively these heterogeneous features can be integrated. Three commonly used fusion strategies are considered in this study.

- Concatenation: The global and local features are concatenated along the channel dimension, which preserves the feature information from both branches in a relatively complete manner. This strategy provides richer representations for subsequent processing, but it also increases the feature dimensionality, resulting in additional parameters and computational overhead.
- Element-wise Addition: The two feature streams are fused by direct summation. This strategy is computationally lightweight and does not change the feature dimension. However, its simple linear combination may weaken branch-specific information and lead to the loss of some discriminative details.
- Element-wise Multiplication: The two branches are fused by element-wise multiplication, which strengthens interactive responses between the corresponding features. This operation can emphasize commonly important patterns and suppress irrelevant activations. On the other hand, if the suppression effect is too strong, some useful complementary information may also be removed.

The recognition accuracy, parameter count, and computational cost of different fusion methods are presented in Table 4. The results show that the fusion strategy has a noticeable effect on the performance of the dual-branch framework. Among the fixed fusion methods, concatenation achieves an accuracy of 96.62%, while both addition and multiplication improve the accuracy to 96.83%, indicating that direct feature interaction is more effective than simple feature stacking.

**Table 4:** Comparison of different branch fusion methods.

Fusion method	Accuracy (%)	Params (M)	FLOPs (G)
Concat	96.62	4.03	1.108
Addition	96.83	4.03	1.108
Multiplication	96.83	4.03	1.108
**Adaptive fusion**	**98.29**	**4.49**	**1.108**

Bold values indicate the results of the proposed method or proposed component.

More specifically, concatenation preserves the information from both branches, but it does not explicitly model their complementary relationships. Addition provides a simple and efficient fusion scheme with unchanged model complexity, although its linear combination may weaken some discriminative branch-specific information. Multiplication further enhances the interaction between the two branches by emphasizing commonly activated responses, but as a fixed operation, it still cannot adaptively adjust the relative importance of the two feature streams.

In contrast, the proposed Adaptive Fusion module achieves the highest accuracy of 98.29%. This improvement suggests that the local and global branches do not contribute equally for all gestures, and an adaptive weighting mechanism is beneficial for exploiting their complementary information. Moreover, the bilinear interaction branch further captures second-order correlations between the two feature streams, which cannot be represented by simple linear fusion. Although the parameter count increases from 4.03 M to 4.49 M, the FLOPs remain unchanged at 1.108 G, indicating that the performance gain is obtained with limited additional model complexity.

#### 4.3.4 Comparison with other methods

In this subsection, we select several representative and state-of-the-art neural networks as baseline models. Specifically, Mekruksavanich et al. (2023) proposed a four-stage cascaded CNN model designed for UWB radar gesture recognition, where each stage comprises a 3 × 3 convolutional layer for feature extraction, followed by a max-pooling layer for dimensionality reduction and a dropout layer to enhance generalization capability. DIAT-RadSATNet (Kumawat et al., 2022) employs a customized Residual Squeeze and Expand (RsE) module alongside an intermediate-layer downsampling strategy to effectively balance recognition accuracy and computational complexity. Khan and Kwon (2025) utilizes an early fusion technique to integrate multi-view UWB radar signals, employing a CNN for spatial feature extraction, LSTM networks for temporal sequence modeling, and an attention mechanism to filter out background noise and irrelevant signals. Additionally, STNet (Luo et al., 2023) extracts spatio-temporal features through convolutions and Temporal Convolutional Networks (TCN), while Fhager et al. (2019) applies a pre-trained ResNet-50 model to the classification of radar range-time spectrograms.

As shown in Table 5, MV-GLFNet achieves the best overall performance among the compared methods, with an accuracy of 98.29%, a precision of 98.32%, a recall of 98.29%, and an F1-score of 98.29%. These results indicate the effectiveness of the proposed global–local fusion framework for UWB gesture recognition. In particular, the collaborative modeling of fine-grained local features and global contextual dependencies, together with the adaptive fusion mechanism, contributes to more discriminative spatiotemporal feature representations.

**Table 5:** Performance comparison of MV-GLFNet with other models.

Model	Accuracy (%)	Precision (%)	Recall (%)	F1-score (%)
Mekruksavanich et al. (2023)	96.58	96.70	96.58	96.54
DIAT-RadSATNet	97.67	97.74	97.67	97.64
Khan and Kwon (2025)	95.79	95.93	95.79	95.75
STNet	97.58	97.61	97.58	97.56
Fhager et al. (2019)	96.92	97.03	96.92	96.88
**MV-GLFNet (ours)**	**98.29**	**98.32**	**98.29**	**98.29**

Bold values indicate the results of the proposed method or proposed component.

To further analyze the behavior of the proposed framework, we examine the effect of different input viewpoints on recognition performance. As shown in Table 6, the recognition performance is clearly affected by the input viewpoint configuration. Since the TR representations captured by IR-UWB radar vary with the observation direction, three input settings are considered in this study, namely single-view input, dual-view combinations, and tri-view fusion.

**Table 6:** Accuracy (%) of different methods under various input-view settings.

Method	Left	Right	Top	Left + right	Left + top	Right + top	Left + right + top
Mekruksavanich et al. (2023)	86.50	77.21	68.71	88.00	96.00	95.13	96.58
DIAT-RadSATNet	84.00	75.62	67.00	87.85	96.00	96.33	97.67
Khan and Kwon (2025)	79.63	77.79	65.12	78.25	93.71	93.29	95.79
STNet	86.50	77.58	71.29	90.08	96.13	97.38	97.58
Fhager et al. (2019)	81.75	71.96	67.08	85.54	94.75	95.75	96.92
MV-GLFNet	**88.13**	**81.29**	**77.82**	**91.06**	**95.12**	**96.71**	**98.29**

Bold values indicate the results of the proposed method or proposed component.

For the single-view setting, the Left view consistently outperforms the Right and Top views, whereas the Top view yields the lowest standalone accuracy. This result is mainly attributed to the view-dependent nature of IR-UWB TR representations. Since a TR map records range-dependent echo variations along the radar line-of-sight, gestures with larger radial motion components produce clearer and more discriminative energy trajectories. Under the adopted radar configuration, the Left view provides more salient range-varying responses for several directional and diagonal gestures, while the Top view observes some gestures with relatively limited radial displacement, leading to weaker or more compressed TR patterns. The difference between the Left and Right views may also be related to asymmetric gesture execution, view-dependent scattering, and possible self-occlusion effects.

Although the Top view performs worst when used alone, it is not redundant. Instead, it provides complementary information to the side views. As shown in Table 6, dual-view combinations generally outperform single-view inputs, and the tri-view configuration achieves the best performance. This result indicates that multi-view fusion can alleviate viewpoint-specific information loss and provide a more complete representation of gesture dynamics.

In practical deployment, the accuracy improvement from tri-view sensing should be balanced with the added system cost. A three-radar setup requires additional hardware, installation space, view alignment, and possible synchronization or calibration. Thus, it is more suitable for fixed interaction scenarios where robustness to viewpoint variation is important. For cost- or space-limited applications, the single-view and dual-view results in Table 6 provide practical alternatives with lower system complexity.

**Figure 9:**
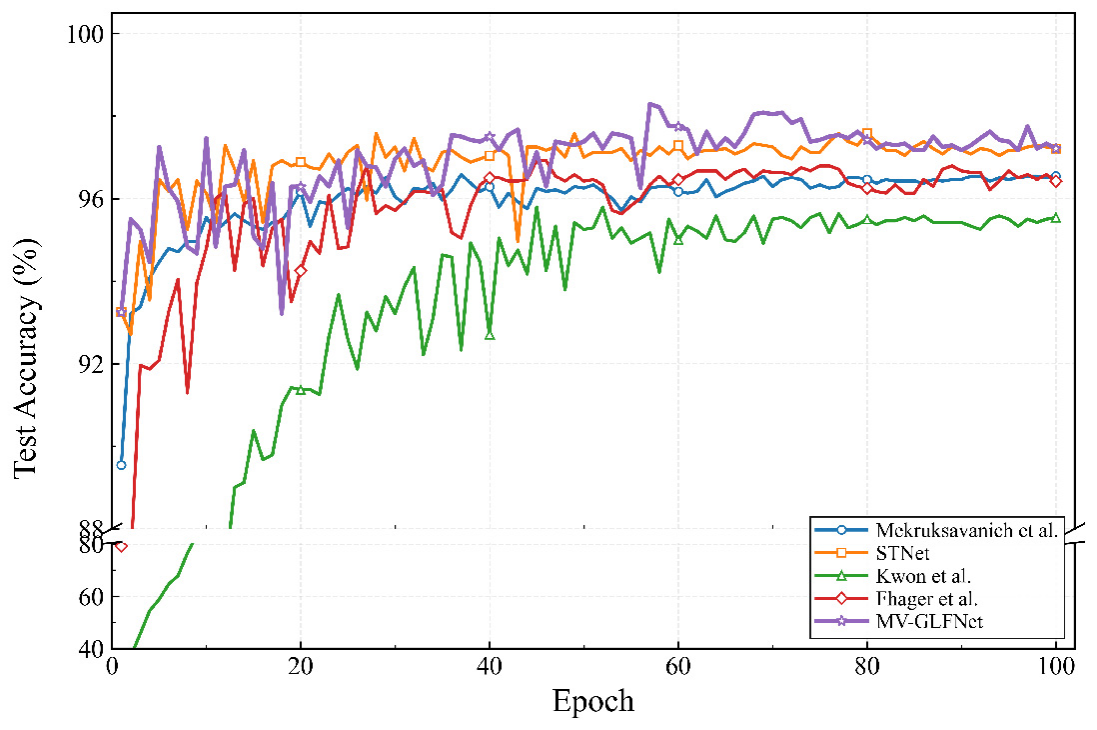
Comparison of test accuracy curves for different models over training epochs.

After analyzing the effect of input views, we further evaluate the convergence behavior of different models by monitoring the test accuracy over training epochs. As shown in Figure 9, MV-GLFNet converges rapidly and attains high accuracy within a few epochs. Although STNet also shows competitive performance, its final accuracy and curve smoothness remain slightly inferior to those of MV-GLFNet. This observation indicates that MV-GLFNet achieves more stable training behavior and better recognition performance.

**Figure 10:**
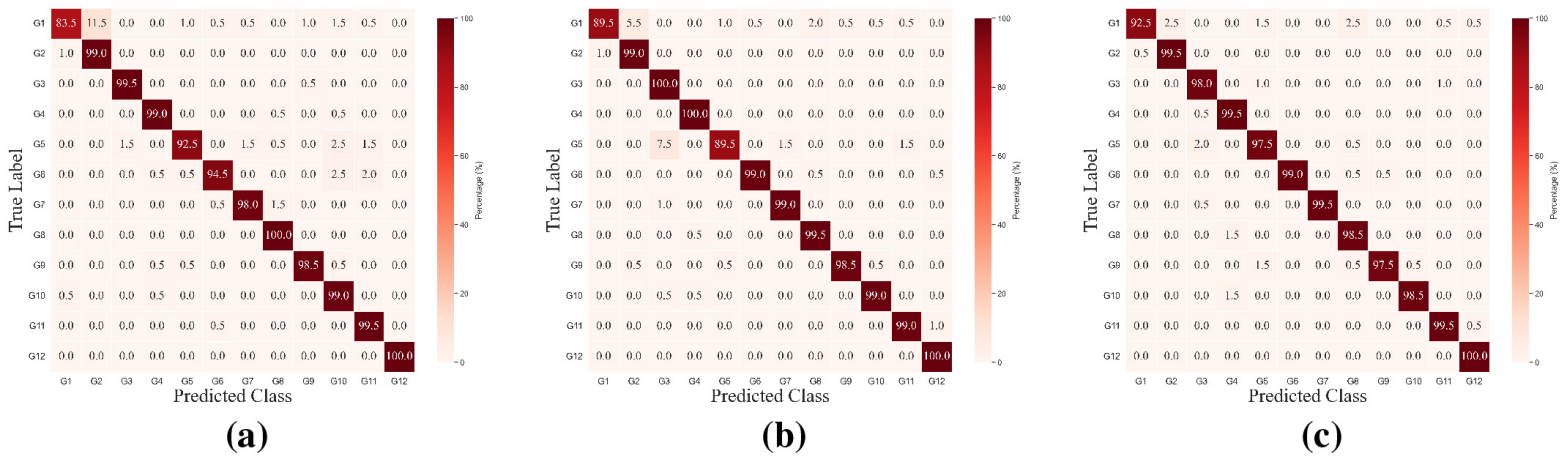
Confusion matrix for ablation experiments. **(a)** Local Backbone, **(b)** Global Backbone, and **(c)** MV-GLFNet.

**Figure 11:**
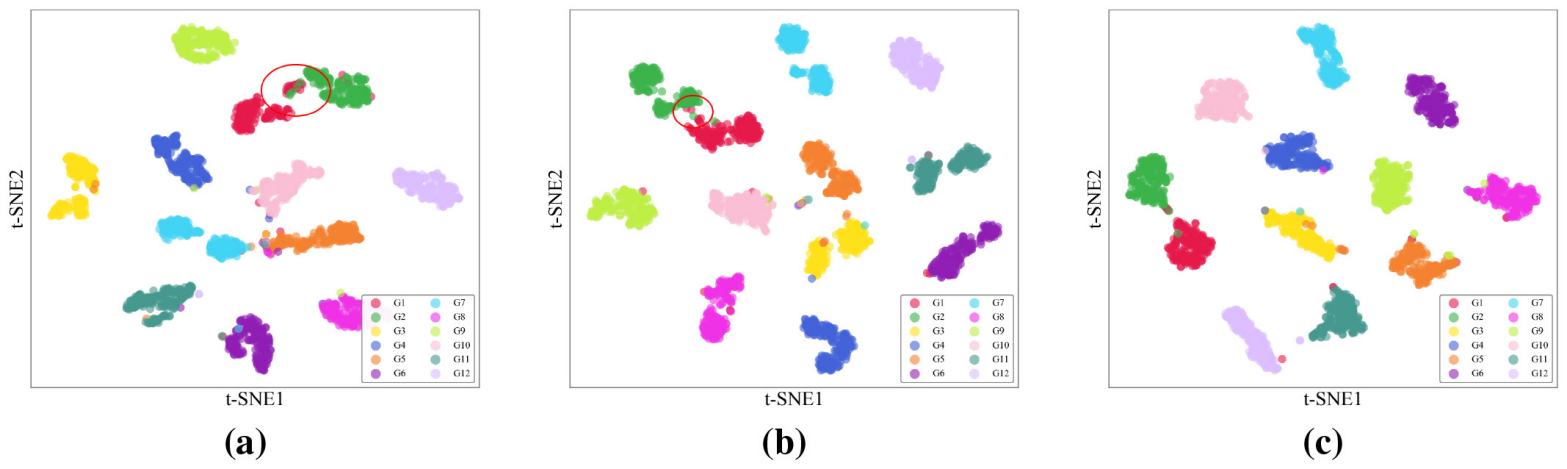
t-SNE for ablation experiments, red circles indicate the overlap between G1 and G2. **(a)** Local Backbone, **(b)** Global Backbone, and **(c)** MV-GLFNet.

Figures 10, 11 present the confusion matrices and t-distributed stochastic neighbor embedding (t-SNE) (van der Maaten and Hinton, 2008) visualizations of the Local Backbone, Global Backbone, and MV-GLFNet, respectively. The confusion matrices reflect class-wise prediction errors, while the t-SNE results provide a feature-space interpretation of these errors.

The Local and Global Backbones correctly classify most gesture categories, but confusion remains for highly similar directional gestures, especially G1 and G2. Such gestures may exhibit comparable range distributions and energy trajectories in the TR domain, with discriminative cues mainly contained in subtle local trajectory variations and temporal evolution. Therefore, relying only on local texture modeling or global temporal modeling may lead to insufficient separation between these classes, as reflected by the off-diagonal responses in Figures 10a, b, and the adjacent clusters in Figures 11a, b.

In contrast, MV-GLFNet yields a clearer diagonal pattern in the confusion matrix and more compact, better-separated clusters in the t-SNE space. In particular, the feature overlap between G1 and G2 is markedly reduced. These results indicate that the proposed global-local fusion strategy improves the representation of both fine-grained TR motion details and long-range temporal context, leading to stronger intra-class compactness and inter-class separability.

Overall, these results indicate that the Local Backbone is more effective at capturing local detail information, while the Global Backbone is better suited for modeling global structural relationships. By fusing global and local features, MV-GLFNet further improves intra-class compactness and inter-class separability in the feature space, thereby achieving more stable and superior recognition performance across all categories.

**Figure 12:**
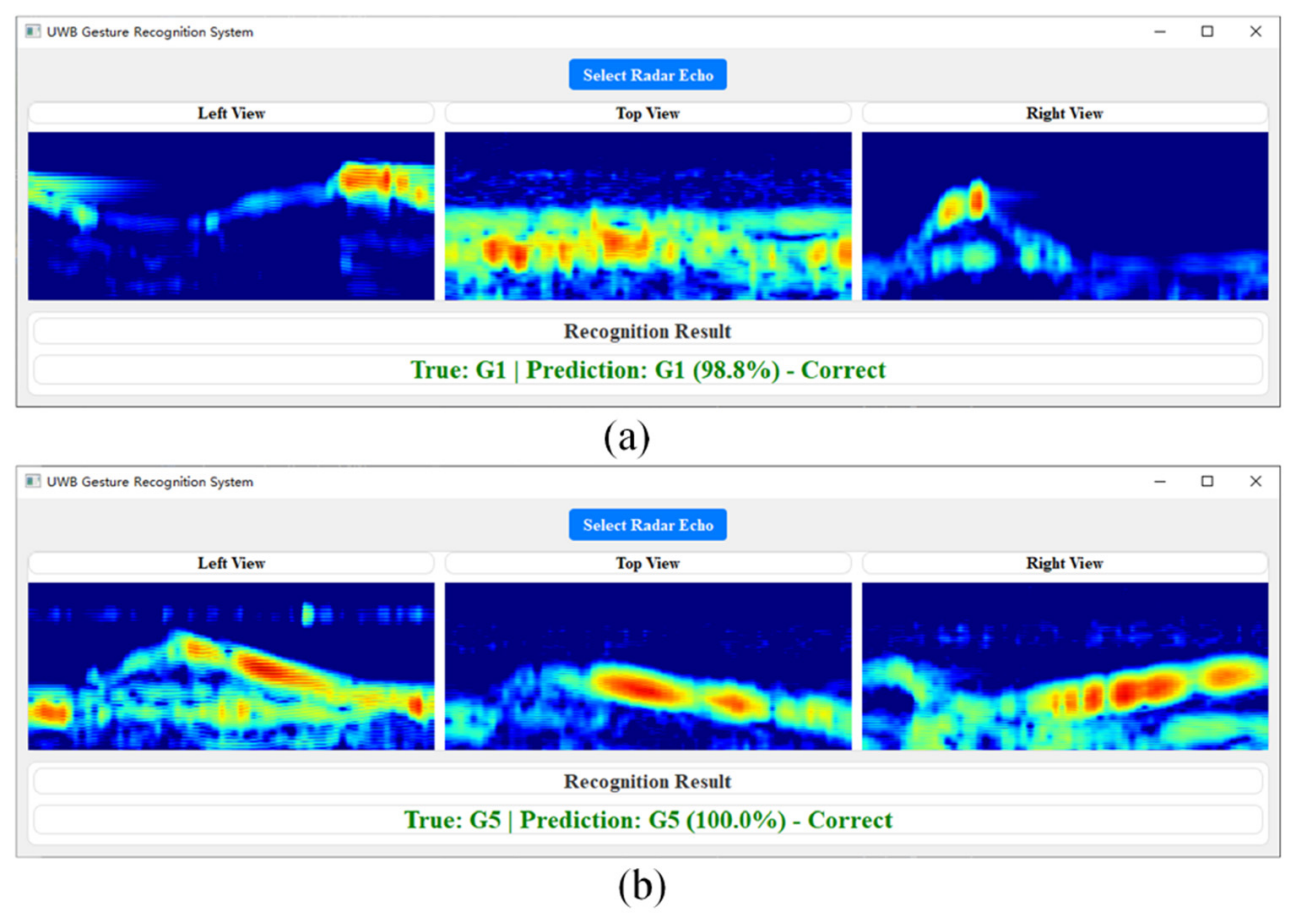
Illustration of the multi-view UWB gesture recognition interface based on MV-GLFNet. **(a)** Correctly classified G1 sample with 98.8% confidence. **(b)** Correctly classified G5 sample with 100.0% confidence.

To further illustrate a potential application scenario of the proposed method, Figure 12 presents an example interface of the multi-view UWB gesture recognition pipeline based on MV-GLFNet. For each input sample, TR maps from the left, top, and right views are displayed simultaneously, together with the ground-truth label, predicted label, and confidence score. This visualization provides an intuitive demonstration of the inference process of MV-GLFNet within a complete multi-view gesture recognition framework. Although the experiments in this study are conducted on a public dataset, this interface example still indicates the potential applicability of the proposed method to practical multi-view UWB gesture recognition scenarios.

## 5 Conclusion

In this work, we presented MV-GLFNet, a neuromorphic-inspired multi-view global-local fusion framework for IR-UWB radar dynamic gesture recognition. To address the limitations of single-view sensing and the insufficient joint modeling of local motion details and global temporal dependencies, the proposed method first exploits motion-enhanced TR maps from three complementary viewpoints through early fusion to improve the spatial completeness of radar observations. On this basis, a dual-branch architecture is designed to capture fine-grained local dynamic textures and long-range global temporal structures in a coordinated manner. Furthermore, an adaptive fusion module that combines gated first-order fusion with bilinear second-order interaction is introduced to enhance feature collaboration and produce more discriminative fused representations.

Extensive experiments on a public 12-class UWB gesture dataset under a subject-independent protocol demonstrate the effectiveness of the proposed framework. The results show that MV-GLFNet achieves an average recognition accuracy of 98.29%, outperforming several representative baseline methods. In addition, the ablation studies verify the contributions of the local backbone, the global backbone, and the adaptive fusion strategy, while the multi-view analysis further confirms that complementary observations from different viewpoints are beneficial for robust gesture recognition. The confusion-matrix and t-SNE visualizations also indicate that the proposed method improves intra-class compactness and inter-class separability, especially for confusing gesture categories.

It should be noted that the present study is neuromorphic-inspired rather than a fully spiking or hardware-level neuromorphic implementation. The neuromorphic relevance of the proposed framework mainly lies in its motion-sensitive representation, hierarchical global-local processing, and adaptive multi-path feature integration. Although the current results are encouraging, several limitations remain. First, the proposed method is validated on a single public dataset, and its cross-dataset and cross-device generalization ability still requires further investigation. Second, the multi-view setting improves recognition performance but also introduces additional sensing and deployment complexity, which should be considered in practical applications.

In future work, we will further investigate lightweight model design, cross-domain generalization, and more deployment-oriented implementations for practical radar-based interaction systems. We will also explore event-driven or spiking extensions of the current framework to further strengthen the connection between radar perception and neuromorphic computing.

## Data Availability

The original contributions presented in the study are included in the article/supplementary material, further inquiries can be directed to the corresponding author.
